# Factors influencing implementation of a patient decision aid in a developing country: an exploratory study

**DOI:** 10.1186/s13012-017-0569-9

**Published:** 2017-03-21

**Authors:** Wen Ting Tong, Yew Kong Lee, Chirk Jenn Ng, Ping Yein Lee

**Affiliations:** 1 Department of Primary Care Medicine, Faculty of Medicine, University of Malaya, Kuala Lumpur, Malaysia; 2 Department of Family Medicine, Faculty of Medicine and Health Sciences, University Putra Malaysia, Serdang, Malaysia

**Keywords:** Implementation, Insulin patient decision aid, Developing countries, Insulin initiation, Type 2 diabetes mellitus, Decision support, Barriers, Facilitators, Factors

## Abstract

**Background:**

Most studies on barriers and facilitators to implementation of patient decision aids (PDAs) are conducted in the west; hence, the findings may not be transferable to developing countries. This study aims to use a locally developed insulin PDA as an exemplar to explore the barriers and facilitators to implementing PDAs in Malaysia, an upper middle-income country in Asia.

**Methods:**

Qualitative methodology was adopted. Nine in-depth interviews (IDIs) and three focus group discussions (FGDs) were conducted with policymakers (*n* = 6), medical officers (*n* = 13), diabetes educators (*n* = 5) and a nurse, who were involved in insulin initiation management at an academic primary care clinic. The interviews were conducted with the aid of a semi-structured interview guide based on the Theoretical Domains Framework. The interviews were audio-recorded, transcribed verbatim and analyzed using a thematic approach.

**Results:**

Five themes emerged, and they were lack of shared decision-making (SDM) culture, role boundary, lack of continuity of care, impact on consultation time and reminder network. Healthcare providers’ (HCPs) paternalistic attitude, patients’ passivity and patient trust in physicians rendered SDM challenging which affected the implementation of the PDA. Clear role boundaries between the doctors and nurses made collaborative implementation of the PDA challenging, as nurses may not view the use of insulin PDA to be part of their job scope. The lack of continuity of care might cause difficulties for doctors to follow up on insulin PDA use with their patient. While time was the most commonly cited barrier for PDA implementation, use of the PDA might reduce consultation time. A reminder network was suggested to address the issue of forgetfulness as well as to trigger interest in using the PDA. The suggested reminders were peer reminders (i.e. HCPs reminding one another to use the PDA) and system reminders (e.g. incorporating electronic medical record prompts, displaying posters/notices, making the insulin PDA available and visible in the consultation rooms).

**Conclusions:**

When implementing PDAs, it is crucial to consider the healthcare culture and system, particularly in developing countries such as Malaysia where concepts of SDM and PDAs are still novel.

## Background

Shared decision-making (SDM) is part of patient-centred care whereby patients and clinicians decide on a treatment together. This is particularly relevant when the decision is preference-sensitive [1]. One way to promote SDM is to use a patient decision aid (PDA), which provides information about the decision, available treatment options, benefits and risks of each option and ways to clarify patient values [2]. In a Cochrane Review of 115 randomized controlled trials, PDAs have been proven to be effective in involving patients in SDM and improving their decision quality [2]. However, the adoption and implementation of PDAs in clinical practice remains poor [3–5]. One study has found that only about 10% of eligible primary care patients actually received PDAs despite clinic-wide PDA adoption [4].

There are many factors that influence the implementation of SDM and PDAs. Studies have highlighted barriers such as time constraints, healthcare professionals’ attitude, perceived legitimacy of the PDA, lack of applicability due to patient characteristics, clinic capacity, processes of care and the healthcare environment [6–8]. Among the facilitators were provider’s motivation, provision of training and skills development for providers, identification of a clinical champion, introduction of a system to identify eligible patients to use PDAs ahead of clinical consultations, positive impact on the clinical process and patient outcomes [7, 8]. However, the relative influence of these factors varies across different countries.

As most of the studies on barriers and facilitators to implementation of PDAs are conducted in the west [6–8], the findings may not be transferable to Asian countries, most of which are developing countries, and issues such as socio-cultural barriers (language barrier and physician paternalism) and lack of resources (infrastructure or technology development) may be more significant compared to developed countries. Nevertheless, studies have shown that patients in Asia want to be involved in SDM [9, 10] and there are an increasing number of PDAs being developed in the region [11, 12]. However, no Asian studies have reported the implementation of these PDAs.

Malaysia is a developing upper middle-income country in Southeast Asia [13]. It has a population of 28.3 million, comprising three main ethnic groups namely Malay (67.4%), Chinese (24.6%) and Indian (7.3%) [14]. The implementation of SDM in Malaysia is hampered by several factors including the multi-cultural and language diversity of the population and the lack of patient involvement in healthcare [15]. In Malaysia, although the national language is the Malay language, not all Chinese or Indian patients are fluent in this language. Furthermore, each ethnic group has their own cultural norms and beliefs when it comes to health. Thus, doctors and patients may be mismatched linguistically as well as culturally. As an attempt to advance SDM, several Malaysian PDAs have been developed for patients who are making a decision on insulin use [12], early breast cancer [16] and early prostate cancer treatment [17]. These PDAs are available in the Malay, Chinese, Tamil and English language to facilitate decision-making as they will be able to read and understand the PDA in their preferred language. Furthermore, these PDAs explored cultural beliefs of the major ethnicities in Malaysia. Nevertheless, formal implementation of these PDAs has not been conducted. Therefore, this study aims to use a locally developed insulin PDA (http://dmit.um.edu.my/?modul=DMIT_PDA) [12, 18] as an exemplar to explore the barriers and facilitators to implementing PDAs in a developing country.

## Methods

### Study design

This study adopted a qualitative methodology to explore the factors influencing implementation of the insulin PDA. In-depth interviews (IDIs) and focus group discussions (FGDs) were conducted.

### Study setting and participants

Data collection was carried out at an academic primary care clinic at the University of Malaya Medical Center, an urban government teaching hospital. In Malaysia, the majority of diabetes patients are managed in the public sector where healthcare services are subsidized by the government. The primary care clinic is an outpatient clinic which accepts patients from all over Malaysia, and therefore, it has a high patient load with patients from various backgrounds. On average, there are 25 doctors in the clinic, two diabetes educators (DE) and 26 registered nurses (RN). Generally, patients in the clinic do not get to decide which doctor to see; hence, they may not consult the same doctor during the follow up.

There are three groups of healthcare professionals involved in insulin initiation in the clinic: doctors, DEs and RNs. In the clinic, only doctors (i.e. family medicine specialists) and medical officers were allowed to prescribe insulin to patients. There is a diabetes education centre located in the clinic where three DEs, who are nurses, provide patient education on diabetes, blood glucose monitoring and insulin initiation and injection techniques after receiving referrals from doctors. To qualify as a DE, RNs need to complete an advanced diploma in diabetes education. In the course, the RNs are trained on diabetes education, treatment and nursing of diabetes patients (including on insulin initiation). However, training on SDM and PDA is not part of the curriculum as the insulin PDA has not been formally introduced or implemented in the healthcare system in Malaysia. In contrast to DEs, RNs’ tasks are to assist doctors in the consultation rooms. Of 26 individuals who were approached to participate in the study, one staff nurse refused due to time constraints.

This study used purposive sampling to recruit participants who were healthcare policymakers (HPMs) and healthcare providers (HCPs) working in the medical centre [19]. The inclusion criteria for HPM were those who were responsible for or involved in making decisions on whether a particular health intervention should be implemented in the hospital, while for HCPs were those who were involved in advising patients about insulin initiation. The HPMs recruited included the director-level hospital manager, Endocrine and Primary Care Medicine policymakers and hospital matrons. They set standards of care and implement programmes to improve diabetes care in the hospital. While they perform managerial tasks, they are also practicing HCPs. HCPs included the doctors, DEs and RNs. In order to achieve maximum variation, we recruited participants from different socio-demographic backgrounds (ethnicity, gender) and included those with and without experience in using the PDA. The PDA was previously pilot-tested with a small group of HCPs in the clinic to test its acceptability, and a few of them had continued using it after the pilot-testing phase had ended. HPMs were not involved in the pilot study.

### Study instrument

The interviews were conducted with the aid of a semi-structured interview guide (Table 1), which was developed based on the Theoretical Domains Framework (TDF) [20] and a literature review on barriers and facilitators to implementing PDAs [7]. The TDF is an overarching framework of 14 theoretical domains synthesized from behaviour change constructs found in 33 behaviour change theories (Table 2). The TDF was selected as the theoretical basis for this study because implementation success is largely dependent on the behaviour change of the users involved on whether to adopt or reject the implementation innovation. The TDF is comprehensive as it not only looks into the impact of rational and cognitive process of an individual but also looks into emotional factors [21] as well as external, organizational factors such as working environment and resources that may influence them on whether or not to adopt a new behaviour or innovation such as the practice of SDM and use of PDA. Furthermore, the TDF has been used in many clinical behaviour change implementation research projects [22], which is why it was felt to be appropriate for this study to understand the factors influencing implementation of insulin PDA.

**Table 1:** Summary of the study interview guide

Preamble: We would like to implement the insulin PDA in the clinic. We would like to hear your honest opinion on what are the barriers and facilitators to implementing the insulin PDA in your healthcare organization so that it is effective and sustainable to be used.
Insulin PDA • What do you think about the insulin PDA? (Probe: feelings: afraid, hopeful) (emotions) Would you use this PDA? (intention, beliefs about consequences, optimism)
Individuals • Do you think that HCPs’ will want to use the insulin PDA? Why? (intentions) • Do you think that insulin PDA will affect your/HCP role? How? (social professional role and identity) • Do you think that you/HCPs would remember to use the PDA if it is implemented? Why? Why not? (memory, attention and decision processess) • Do you think that you/HCPs will be confident to use the insulin PDA? Why? (beliefs about capabilities) • How doctors and nurses or other HCPs such as dieticians or pharmacists play a role in implementing the insulin PDA? (social professional role and identity) • Do you think that you/HCPs have the knowledge and skills to use the insulin PDA? What are the knowledge and skills needed? (knowledge and skills) • Do you see any benefit or harm in implementing the PDA in the current healthcare system? (beliefs about consequences) • Who do you think can influence you/HCPs whether to use or not to use the insulin PDA? How? (social influences) • What can you/HCPs do to use the insulin PDA in their consultation? (behavioural regulation) • If you will implement the insulin PDA, what are the goals you/patients want to achieve? (goals)
Inner context – the clinic • Does your institution have the resources to implement the insulin PDA? What are the resources available and what are needed? • How do you think your organization’s working culture (general beliefs, values, assumptions that people embrace, receptivity of a new intervention) will affect the implementation of the PDA? (environmental context and resources)
Outer context – the healthcare system • Are you confident/positive/optimistic that the current healthcare system is capable to successfully implement the insulin PDA despite any difficulty? Yes/No, why? (beliefs about capabilities; optimism) • What can be done by the higher-level authority to ensure the success of the implementation of the PDA? (reinforcements) • In your opinion, how can we make the implementation of the insulin PDA sustainable?

**Table 2:** TDF [20]

	Domain	Definition (American Psychological Associations’ Dictionary of Psychology)
1	Knowledge	An awareness of the existence of something
2	Skills	An ability or proficiency acquired through practice
3	Beliefs about capabilities	Acceptance of the truth, reality or validity about an ability, talent or facility that a person can put to constructive use
4	Optimism	The confidence that things will happen for the best or that desired goals will be attained
5	Beliefs about consequences	Acceptance of the truth, reality, or validity about outcomes of a behaviour in a given situation
6	Reinforcement	Increasing the probability of a response by arranging a dependent relationship, or contingency, between the response and a given stimulus
7	Intentions	A conscious decision to perform a behaviour or a resolve to act in a certain way
8	Goals	Mental representations of outcomes or end states that an individual wants to achieve
9	Memory, attention and decision processes	The ability to retain information, focus selectively on aspects of the environment and choose between two or more alternatives
10	Emotion	A complex reaction pattern, involving experiential, behavioural and physiological elements, by which the individual attempts to deal with a personally significant matter or event
11	Social/professional role and identity	A coherent set of behaviours and displayed personal qualities of an individual in a social or work setting
12	Environmental context and resources	Any circumstance of a person’s situation or environment that discourages or encourages the development of skills and abilities, independence, social competence, and adaptive behaviour
13	Social influences	Those interpersonal processes that can cause individuals to change their thoughts, feelings, or behaviours
14	Behavioural regulation	Anything aimed at managing or changing objectively observed or measured actions

### Data collection process

HPMs and HCPs who fulfilled the study inclusion criteria were invited to participate in the study. Prior to the interviews, eligible participants who consented to participate in the study were given information about the insulin PDA, its objective and content, the concept of SDM and the various modalities (booklet, tablet, website) available. A video, which demonstrates how the insulin PDA can be used during a consultation, was shown to the participants to give them an idea on how the insulin PDA can be used in the clinic. The participants were also encouraged to think about other ways of implementing the insulin PDA in their practice.

The interviews were conducted between December 2015 and March 2016 by the researchers (WTT, YKL, CJN) who asked the participants ‘open’ and ‘probing’ questions to explore the factors influencing implementation of the insulin PDA in their practice. All the interviews were face-to-face and were conducted in the clinic consultation rooms during rest times in between clinical consultations, or at the office of the participants. The interviews lasted from 50 to 90 min. This study was conducted in the university hospital setting, and three of the researchers (WTT, YKL, CJN) were known to the participants as they are based at this university. WTT is a PhD student, YKL is a lecturer, while both CJN and PYL are clinical lecturers who specialize in family medicine. All researchers are experienced in conducting qualitative research. As WTT did not know any of the participants prior to the interviews, WTT conducted most of the interviews (8/12) as YKL and CJN refrained from interviewing participants whom they knew. PYL did not conduct any interview. The interviews were audio-recorded, and field notes were written to document the content of and reflections on the interviews. The data collection ceased when data saturation was achieved; that is when the barriers or facilitators that emerged from the data became repetitive and there were no more new findings.

### Data analysis

All the interviews were transcribed verbatim and checked for accuracy before being imported into Nvivo software for data analysis. The researchers (WTT, YKL, CJN, PYL) familiarized themselves with the first three transcripts and coded the transcripts independently using a thematic approach. The transcripts were read and coded line by line. A code is a short text which represents the meaning of the text segment. Codes that have similar meaning were grouped together to form a category, and later the categories were compared and merged into bigger themes [23]. Researchers met to discuss the categories and themes which emerged from their individual analysis. Any discrepancies in the categories and themes were resolved through consensus before finalizing the coding framework, which was used to analyze the remaining transcripts by one of the researcher (WTT). Any new codes and categories that emerged were added to the list of themes and categories upon consultation with the research team. The written field notes and interview reflections were triangulated with the results to ensure that the findings were correctly interpreted and no information were missed out and to supplement the interview findings. By comparing data from multiple sources, we hope to enhance the credibility of the findings [24].

### Ethics approval

This study received ethics approval from the University of Malaya Medical Centre Medical Ethics Committee (reference: MECID.NO: 20158-1600).

## Results

### Socio-demographic and practice profile of participants

Nine IDIs and three FGDs were conducted with 25 individuals who participated in the study: the hospital policymakers (*n* = 6), medical officers (*n* = 13), DEs (*n* = 5) and a RN. Two of the FGDs were conduced with medical officers (*n* = 7; *n* = 6). Another FGD with the nursing policymakers was conducted with smaller number of participants (*n* = 3) as they were the only three senior nursing staff at management level who were felt to be able to provide feedback on the implementation of the insulin PDA. There were six participants who have had experiences in using the insulin PDA (Table 3).

**Table 3:** Characteristics of participants

Interview	Role	Ethnicity	Age	Sex	Experience with insulin PDA
IDI 1_ Diabetes educator 1	Healthcare provider	Chinese	56	Female	No
IDI 2_ Diabetes educator 2	Healthcare provider	Malay	57	Female	Yes
IDI 3_ Diabetes educator 3	Healthcare provider	Malay	53	Female	No
IDI 4_ Diabetes educator 4	Healthcare provider	Malay	35	Female	No
IDI 5_ Diabetes educator 5	Healthcare provider	Malay	57	Female	No
IDI 6_ Registered nurse	Healthcare provider	Indian	55	Female	No
IDI 7_ Primary care policymaker	Healthcare policymaker	Malay	48	Female	No
IDI 8_ Endocrine policymaker	Healthcare policymaker	Chinese	37	Male	No
IDI 11_ Director-level hospital manager	Healthcare policymaker	Malay	43	Male	No
FGD 1_ Medical officer 1	Healthcare provider	Malay	35	Female	No
FGD 1_ Medical officer 2	Healthcare provider	Chinese	32	Female	No
FGD 1_ Medical officer 3	Healthcare provider	Indian	37	Female	Yes
FGD 1_ Medical officer 4	Healthcare provider	Indian	35	Male	No
FGD 1_ Medical officer 5	Healthcare provider	Malay	32	Male	No
FGD 1_ Medical officer 6	Healthcare provider	Malay	32	Male	No
FGD 1_ Medical officer 7	Healthcare provider	Indian	39	Male	Yes
FGD 2_ Medical officer 8	Healthcare provider	Malay	31	Female	Yes
FGD 2_ Medical officer 9	Healthcare provider	Malay	33	Female	No
FGD 2_ Medical officer 10	Healthcare provider	Chinese	33	Female	Yes
FGD 2_ Medical officer 11	Healthcare provider	Chinese	35	Female	No
FGD 2_ Medical officer 12	Healthcare provider	Malay	36	Female	No
FGD 2_ Medical officer 13	Healthcare provider	Malay	36	Female	Yes
FGD 3_ Hospital matron 1	Healthcare policymaker	Malay	56	Female	No
FGD 3_ Hospital matron 2	Healthcare policymaker	Malay	56	Female	No
FGD 3_ Hospital matron 3	Healthcare policymaker	Chinese	57	Female	No

### Emerging themes

Five themes emerged from the interviews, and they were lack of SDM culture, role boundary, lack of continuity of care (COC), impact on consultation time and reminder network.

- Theme 1: Lack of SDM culture
  The participants highlighted that there is a lack of SDM culture in the current practice as HCPs’ paternalistic attitudes still prevailed whereby doctors would make treatment decision for patients and did not like patients to ask too many questions. In addition, patients tended to play a passive role in decision-making and trusted the physicians to make the decision for them. This rendered the lack of need for SDM and use of the insulin PDA.
  I think in our Asian context, we still have this idea of doctor telling you what to do. I think this shared decision approach is a concept that developed countries have but I don’t know whether our culture has reached this stage or not.//Our practitioners don’t give patients chance to decide on their own. We like to tell the patient what to do. So they don’t see the need for this book. The patients still have the mindset of ‘You tell me what to do. It’s not my decision, it is your decision, I just follow you’.//I don’t know whether our patient is up to that yet for the shared decision part. – IDI 1_DE 1_have not used insulin PDA
  Because we are busy in the clinic, sometimes we don’t like people to ask us a lot of questions. FGD 3_Hospital matron 1_have not used insulin PDA
- Theme 2: Role boundary
  There was a clear role boundary between the doctors and the nurses which made collaborative implementation of the PDAs challenging. All participants, including medical officers, felt that the doctor should be the key person to introduce and use the insulin PDA with patients because the decision to initiate insulin treatment lies within the doctor after reviewing patient’s glycaemic control and health profile. Furthermore, only doctors have the authority to prescribe insulin for patients.
  I think the initiation [to use insulin PDA] has to come from the doctor because we are the ones who will know whether it is appropriate or not to talk about insulin initiation. I think only doctors would understand circumstances such as if a patient has cataract and will not be able to inject herself or have no social support. Rather than at the pharmacy end ‘Oh, your Hba1c is very bad. You should be on insulin, here, take this book’ I think that is not right. FGD 2_Medical officer 8_have used insulin PDA
  But we don’t have so much opportunity to initiate (insulin PDA use) because instructions are passed down from the top, ‘ok you do this’. They (doctors) are the first liner. IDI 1_DE 1_have not used insulin PDA
  However, assigning RNs to use the PDA with patients would not be accepted if it fell outside the scope of RNs’ current duties. For example, when asked if RNs had a role in using the insulin PDA with patients, participants voiced that RNs would not perceive the use of insulin PDA to be part of their job scope. RNs confined their duties only to pre-determined tasks and would not be interested to perform additional tasks. They perceived using the insulin PDA as an added workload.
  But I think because they [RNs] don’t see it as their role to advise patients about their disease. It is more of getting the patient in, sorted out, weight, height. So, I think to get them to start [using insulin PDA] it might be a bit harder than to get the medical officers to use it. – IDI 7_Primary care policymaker_have not used insulin PDA
  Nurses, we only want to do what is nursing. We don’t want to do beyond that. ‘I’m a nurse … why should I do this, this is nothing concerning me, this is doctors’ job, not my job’.//‘Oh another booklet. Another thing to talk to the patient now’. Anything else is added work. – IDI 6_RN_have not used insulin PDA
  As for DEs, some felt that their role in the PDA implementation was just ‘taking orders from doctors’ on when to use the PDA with patients, while some felt that they could inform patients on when to initiate insulin.
  Doctor will say to the patient they have no time [to use PDA] ‘You go and see the DE’. The decision is made by doctor. I just follow order. IDI 2_DE 2_have used insulin PDA
  Actually we also have a clinic of our own and patients come to us we will monitor them. So when we see that they are losing control then we can tell them that ‘I think you need insulin already’. So we also have a little part to play. IDI 1_DE 1_have not used insulin PDA
- Theme 3: Lack of COC
  Some medical officers have continued the insulin PDA use after the pilot study because they felt that the PDA helped to reduce consultation time and address language barrier when they provided information to patients who spoke a different language. However, the use of PDA was later discontinued due to lack of COC. Furthermore, as there was no proper record that the insulin PDA had been given to patients, the medical officer might not be aware that the patient had already been introduced to the insulin PDA by another doctor. This sentiment is shared by both medical officers who have and have not used the PDA.
  Basically, COC is not there. Because doctors don’t get to see the same patients. We might give this PDA to a patient this visit. And next visit, you may not be the one who sees the patient. – FDG 1_Medical officer 4_have not used insulin PDA
  The book was in the diabetic education room. So I did refer patient there so that the nurse can explain but I’m not sure what the nurse explained to the patient. FGD 2_Medical officer 13_have used PDA
  Furthermore, as the setting was a teaching hospital, trainee doctors leave after their training is completed. Therefore, this posed another issue for implementation as doctors who were trained on how to use the insulin PDA may leave and new doctors may not be familiar with the PDA.
  We have to train them (on PDA use), so we have to give them the exposure of what it is like, what patients are like, but you know it takes several consultations to fully get that. They can deploy the book, but then they may not be around next month to even follow up. – IDI 8_Endocrine policymaker_have not used insulin PDA
- Theme 4: Impact on consultation time
  Time constraints were the most commonly raised implementation barrier of the PDA among the participants. Due to high patient load, there were concerns about taking up extra time to go through the insulin PDA with patients during consultations.
  Time is a problem because there are many patients. Whether we have enough time to go through (the insulin PDA) with the patient. – FGD 1_Medical officer 4_have not used insulin PDA
  In contrast, some participants felt that the insulin PDA might help to reduce consultation time. However, this is only if patients used the insulin PDA prior to the clinical consultation such as while waiting for the doctor or at home. It was felt that patients who have read the PDA were better prepared to discuss their concerns with doctors during clinical consultation and this might help to save time.
  This saved time for the doctor. ‘Mr, you read first, what you don’t understand please come back to me’. So he would have read the whole thing. What they didn’t understand they highlight to you and you address. So your time management is shortened. – IDI 6_RN_ have not used insulin PDA
- Theme 5: Reminder network
  The PDA was not integrated into the care pathway, and hence, the HCPs might forget to use it with patients. The participants wanted reminders and suggested different approaches including peer reminder, incorporating the PDA into the clinic electronic medical record (EMR), and displaying posters or PDAs in the clinic to remind both HCPs and patients to use the PDA.
  Doctors, among doctors [to remind one another]. Sometimes the attendants are also assisting the doctors. They may not know how to use it, but they will know there is such a tool.//Nurses who are working in the clinic with the doctors, they can remind the doctor ‘Hey doctor you can use the book you know’. – IDI 1_DE 1_have not used insulin PDA
  It was proposed that the EMR could be programmed to identify patients suitable for using the PDA and to notify the doctors during the consultation. In addition, the electronic copy of the PDA should be made available in the computer’s desktop which can serve as a reminder for the doctor to use the insulin PDA.
  So I suppose what you can do is… if their HbA1c is above certain level, then have the EMR goes alert, ‘PDA, PDA!’. So either I can give the book or I can alert them to go to the website. – IDI 7_Primary care policymaker_have not used insulin PDA
  Every day when we see patients, we have to use the computer. So maybe it should be in the desktop in PDF so that when we see patients, we just open up the PDF. FD4 1_Medical officer 4_have not used insulin PDA
  Another type of reminder is to put up posters or notices about the insulin PDA, as well as making the insulin PDAs available and visible in the consultation room. This will help HCPs remember to use the insulin PDA as well as creating awareness about the insulin PDA among patients.
  Like putting posters in the clinic so that patients or their carers can see. Then patient can remind doctors about the book. – FGD 2_Medical officer 13_have used insulin PDA
  But I think if we alert them more they will use it. So you sort of like, make it available. So I suppose if it is available in the room, and they are reminded all the time. I think it is keep being reminded that there is such a thing. – IDI 7_Primary care policymaker_have not used insulin PDA

## Discussion

This study provides insights into factors influencing implementation of PDAs from a developing country, a setting which is scarcely reported. The factors revealed in our study are not too different from those commonly reported in the western countries such as lack of SDM culture [25–27], time constraints [6, 25] and reminders. Less commonly reported factors which emerged from our study were role boundary and COC. This study highlighted a few important domains from the TDF that could influence SDM implementation in developing countries. For example, under the ‘environmental context and resources domain’, we found that there was low awareness and receptivity to SDM among the HCPs, which then hindered the implementation of PDA in the Malaysian primary care setting.

The participants of this study were doubtful if the concept of SDM is culturally acceptable in Malaysia due to HCP paternalism and patient’s submissiveness towards doctors. This resistance to SDM would hinder the adoption of SDM and subsequently lead to non-use of the insulin PDA. There is a distinct contrast in SDM implementation between developed countries (such as the USA, UK, Germany, Netherlands, Australia, Italy and Spain) where SDM and the use of PDAs have already been implemented at policy and legislation levels [28–31] and developing countries in Asia see slower progress in adopting SDM and PDAs [15, 32]. In Switzerland, however, hierarchical structures and asymmetric physician-patient relationship were reported to be barriers for SDM implementation despite doctors recognizing its importance [31]. This shows similarity to the hierarchical social pattern in Asian culture whereby doctors are placed at a higher societal stratum [33], and patients may therefore tend to consign their healthcare decisions to HCPs. Furthermore, doctors may not wish to offer a choice to patients as it is considered a good practice to initiate insulin in patients with T2DM who are unable to achieve glycaemic control despite taking maximal oral medications. Patients in the public health facilities may feel that they may not have control over their health decision as they only pay a nominal fee (i.e. ‘I get what I pay for’). In addition, they do not have much freedom to choose the HCP they want to see. However, Asian studies have shown that many patients preferred an autonomous (active and shared) role in decision-making [9, 10]. Thus, to facilitate the use of PDAs, the concept of SDM needs to be promoted among HCPs and patients in developing countries. Zhang et al. [34] highlighted that one way to increase patient involvement in making treatment decisions is to increase healthcare professionals’ knowledge about this concept. HCPs also need to be trained on respecting patients’ autonomy and how to engage patients in making decisions about their health care. The Patient Decision Aids Research Group has created continuous education such as online tutorials, interactive workshops, performance feedback and structured protocols in providing decision support [18]. Efforts are also needed to empower patient in being more involved in their healthcare, and one way to do that is to conduct public health campaigns [15]. In developed countries such as Australia and the UK, the ‘AskShareKnow’ [35, 36] and the ‘Ask three questions’ [37, 38] campaigns are conducted to encourage patients to ask their HCPs the three questions (‘What are my options?’, ‘What are the benefits and harms?’ and ‘How likely are these going to happen?’) to increase patient involvement in healthcare. While it has been shown to improve patient-doctor communication in SDM in Australia [35], it is unsure if such campaign would be effective on countries with strong hierarchical social pattern like in developing countries such as Malaysia. Among other strategies that have been proposed to promote SDM in clinical practice, however, at the macro level in Malaysia are the following: (1) incorporation of SDM in clinical practice guidelines, (2) advocating the use of patient decision aids or other decision support tools in patient care, (3) inclusion of patient involvement in decision-making as a quality indicator and (4) payment/reimbursement for practices which implement SDM or use decision aids [15].

Another factor influencing implementation that was raised was role boundary of HCPs in the clinic setting. Role boundary may act both as a facilitator or barrier for the implementation of the insulin PDA. Being clear of one’s job responsibilities helped participants decide if they were the right person to use the insulin PDA. For example, all participants felt that doctors should introduce and use the insulin PDA, given their authority to prescribe insulin and familiarity with the patient’s health profile. Relying solely on doctors would hinder an interprofessional team approach to using the PDA. Involvement of other HCPs besides physicians may help to disperse the work needed in providing decision support. For example, compared to primary care doctors who has to see high number of patients with various conditions, DEs would have more time to provide patient counselling as they only see diabetes patients. Many western studies have also shown HCPs other than doctors, such as nurses, social workers, psychologists and allied health professionals, who play a significant role in ensuring PDAs are implemented successfully by identifying eligible patients, contacting patients about the PDA and providing decision coaching [39–41]. Participants of this current study also suggested that nurses could remind doctors to use the PDA. To increase DEs’ and nurses’ involvement in insulin PDA implementation, they can be trained in decision coaching skills, which have been found to be effective in guiding patients to make an informed decision [42]. SDM and decision coaching training can be included in the advanced diploma in diabetes education and even basic nursing programmes. In addition, doctors also need to be trained to work with the nurses as a team to implement PDA in the clinic. A recent study showed that very few SDM training programmes adopt an interprofessional approach [43], which could help to utilize the strengths of each team members to create a more coordinated and efficient PDA implementation process.

Time constraints are a universal barrier in implementing SDM as well as PDAs [6, 44]. Two studies reported longer consultation time with PDA use compared to usual care [45, 46]. However, some studies indicate otherwise and one strategy that can be used to address the time issue is to highlight to HCPs that the use of PDAs does not always require more time compared to usual care [2, 47, 48] and that informed decision may be achieved more quickly over time [49]. While the first consultation using the PDA may take a longer time, insulin decision-making over subsequent consultations may be shorter, and this reduces delay in decision-making. The participants of this study also felt that pre-visit usage of the insulin PDA might help to save time. Brackett et al. [50] have tested a pre-visit model and found that in addition to time saving, physicians also reported that discussions during a consultation were more focused and helped to address patient values and preferences, which may otherwise be overlooked. It also facilitated decision-making as patients arrived at the clinic appointment more prepared to discuss and make a decision [50]. One strategy used to allay fears over increased time was by asking a mentor or peer expert to demonstrate how the insulin PDA can be incorporated into a standard clinical consultation [51, 52] without taking more time and to emphasize that the delivery of PDA only becomes easier and possibly faster with training and practice.

Another important aspect of the implementation of the insulin PDA is the need to follow up patients (ideally by the same HCP) to ensure that there is continuity in the delivery of the insulin PDA. The use of PDAs is not a one-off event as SDM is a continuous interactive process between HCPs and patients; hence, COC is crucial in ensuring effective delivery of the insulin PDA. The lack of COC in the use of PDAs may cause difficulties for patients to raise or discuss issues pertaining to the PDAs that were brought up in the previous consultation [27]; it also prevents rapport-building between HCPs and patients that could facilitate informed decision-making. In Asia, COC is a challenge in healthcare delivery due to high patient load, lack of manpower, time constraints [53, 54], lack of family physicians and uncoordinated referral mechanisms [55]. COC is also difficult particularly in an academic healthcare setting, where there is a high turnover of staff after the medical trainees have completed their training [53, 56, 57]. To address this barrier, systematic documentation of the use of PDA and its discussions with patients can help to facilitate communication between different doctors who follow up the same patients. Furthermore, new HCPs need to be educated on decision support and PDA use. Such training can be introduced during the orientation programme for new HCPs, having a resource person that provide more information about the PDA in the implementation setting as well as having on-going training and support to familiarize HCPs with the concept of SDM and the use of PDAs.

‘Forgetting to use’ has been reported as a barrier in the implementation of PDAs [58, 59] even after provision of training and continuing medical education credits to encourage use among HCPs [58]. Participants of our study highlighted a reminder network to address the issue of forgetfulness as well as to trigger interest in using the insulin PDA. The use of EMR and IT system has been raised as an effective reminder platform in the implementation of SDM and PDA; for example, they can be used to identify eligible patients for PDAs [44] before their clinic visit, to prescribe PDAs as well as to cue for PDA use [60]. While HCPs have reported they would be more likely to use the PDAs if they were reminded through EMR [4], some felt that an electronic, interactive decision aid linked to a computerized reminder system may not necessarily be better compared to traditional paper resources because of technical issues. The integration of PDA reminders into the EMR will require technology support, which can be a significant barrier in developing countries that are still using paper-based systems [61]. If technology cannot be adopted to facilitate implementation, peer support would be another useful form of reminder, which was also raised by participants in our study. For peer support to be effective, the working culture needs to be one that holds the belief that the use of PDA is the preferred practice style. Efforts are needed to create awareness on the benefits of PDAs so that HCPs will be willing to use them. Physician champion plays an important role in creating awareness about the availability and the importance of PDAs, and encourage the staff to use them; this has been reported as one of the key factors in the successful implementation of PDAs [62]. Another reminder that was raised was having posters and notices to promote the insulin PDA. Promotional brochures placed at exam rooms have been implemented to increase patients’ interest in decision aids [4, 26]. However, while it prompted discussions about PDAs, it did not significantly lead to physicians prescribing PDA to patients [26]. More studies are needed to look at the effectiveness of having promotional materials such as posters or notices on HCPs in remembering to use as well as on patients to initiate discussions with HCPs on PDAs. Ultimately, HCPs and patients need to be made aware that long-term patient engagement in self-management for chronic conditions like diabetes is crucial in improving diabetes control and outcome. This would then obviate the need for reminders. The use of PDA should be integrated as part of the training on longitudinality of care whereby HCPs and patients should be made aware that insulin is an option as diabetes progresses and that PDAs are available to help them make this decision.

### Strengths and limitations of study

This study highlights implementation issues that are pertinent to developing countries where literature on barriers and facilitators to implementation of PDA is scanty. This study explored views from a diverse group of key stakeholders from policymaker, physician, nurse and DE, and this helps to elicit factors influencing implementation from various professional levels.

There are a few limitations in this study. First, there were participants who had not been exposed to or had no experience in using the insulin PDA; they may not be able to grasp the concept of the insulin PDA, which involves the process of shared decision-making between HCP and patients. Instead, they may have thought that the insulin PDA is just another educational material. There is a possibility that some issues pertaining to the implementation processes for PDA may have been omitted. The researchers tried to reduce this limitation by explaining to the participants the purpose of the insulin PDA, concept of SDM, its content, as well as showing a video of a mock consultation using the PDA before conducting the interviews. Second, the data collection was only conducted in an academic primary care clinic and did not include other healthcare settings such as public community health clinics or private practices. Hence, the findings of this study may not be transferable to other settings. Third, it is noteworthy that there was a preponderance of female participants in this study, as more female than male candidates are interested to pursue family medicine as a specialty in Malaysia. However, the researchers felt that the participants’ sex may not have significant impact on the study findings given that the topic of this study is one that is not gender-sensitive. Fourth, only HPMs’ and HCPs’ views were gathered in this study. Patient’s perspectives should also be taken into account, which are currently being explored in the next phase of this study. Lastly, there is a possibility that participants may have provided positive responses or socially desirable answers, as they did not want to criticize their own practice; researchers tried to offset this bias by reassuring the participants that confidentiality would be kept and that their participation in this study would not affect their work or career.

## Conclusions

When implementing PDAs, it is crucial to consider the healthcare culture and system, particularly in developing countries such as Malaysia. This study highlights that to facilitate the implementation and effective use of PDA, there is a need to change physician’s paternalistic attitude as well as to promote patient empowerment so that the concept of SDM can be embraced. The use of an interprofessional approach and reminders should also be considered when designing strategies for PDAs implementation.

## Abbreviations

COC: Continuity of care
DE: Diabetes educator
FGD: Focus group discussion
HCP: Healthcare provider
HPM: Healthcare policymaker
IDI: In-depth interview
PDA: Patient decision aid
RN: Registered nurse
SDM: Shared decision-making
TDF: Theoretical domains framework
